# Supplementary material for: Comparative Genome Analysis of 33 Chlamydia Strains Reveals Characteristic Features of Chlamydia Psittaci and Closely Related Species
Source: Pathogens. 2020 Oct 28;9(11):899. doi: 10.3390/pathogens9110899 (PMC7694038; doi:10.3390/pathogens9110899)
Supplement: Supplementary file 1 [file pathogens-09-00899-s001.zip › Hölzer et al_FigureS1_upsetr_33strains.pdf]

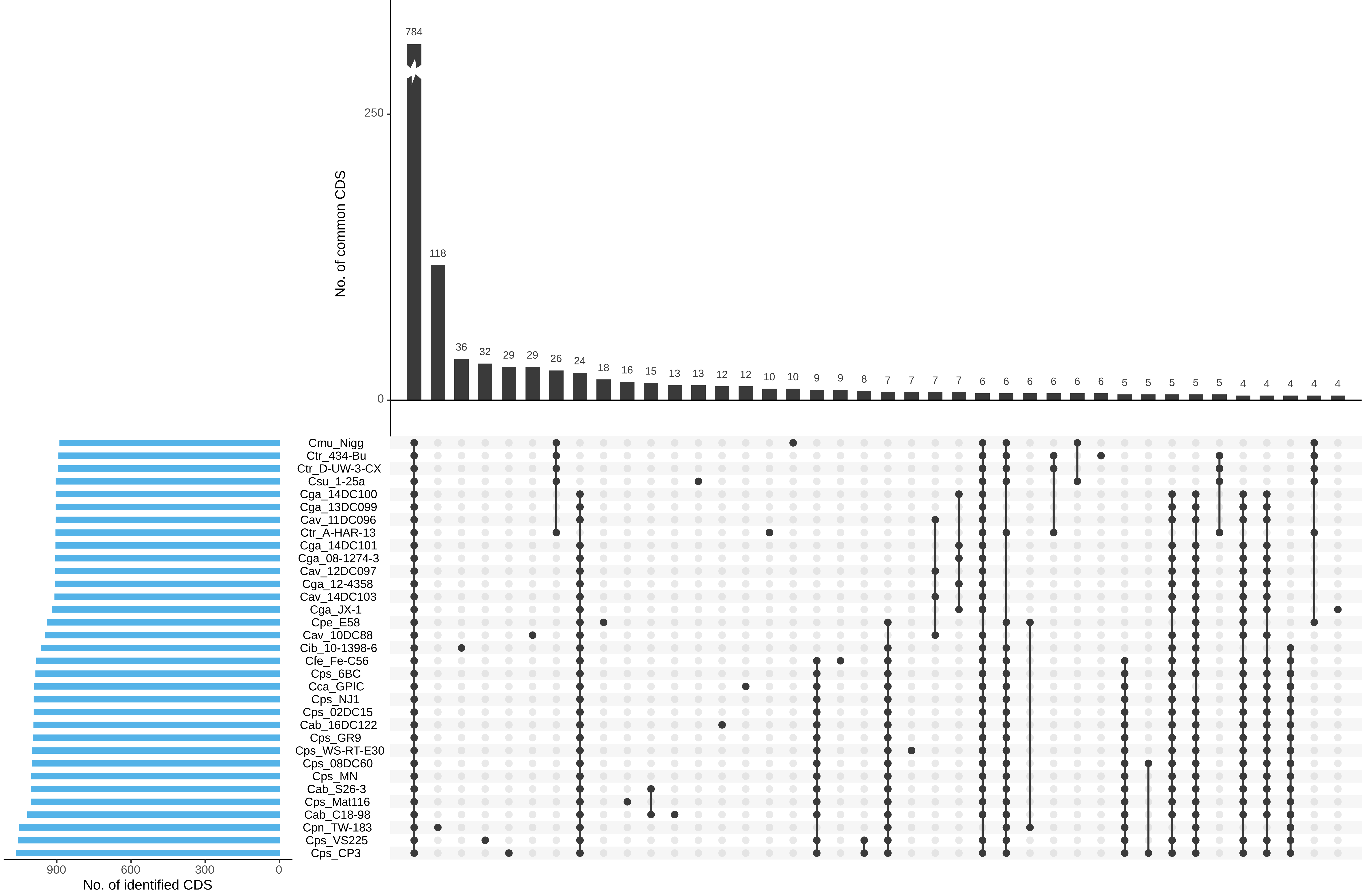

No. of common CDS
784
118
36
32
29
29
26
24
18
16
15
13
13
12
12
10
10
9
9
8
7
7
7
7
6
6
6
6
6
6
5
5
5
5
5
4
4
4
4
4
250
0
Cmu_Nigg
Ctr_434-Bu
Ctr_D-UW-3-CX
Csu_1-25a
Cga_14DC100
Cga_13DC099
Cav_11DC096
Ctr_A-HAR-13
Cga_14DC101
Cga_08-1274-3
Cav_12DC097
Cga_12-4358
Cav_14DC103
Cga_JX-1
Cpe_E58
Cav_10DC88
Cib_10-1398-6
Cfe_Fe-C56
Cps_6BC
Cca_GPIC
Cps_NJ1
Cps_02DC15
Cab_16DC122
Cps_GR9
Cps_WS-RT-E30
Cps_08DC60
Cps_MN
Cab_S26-3
Cps_Mat116
Cab_C18-98
Cpn_TW-183
Cps_VS225
Cps_CP3
900
600
300
0
No. of identified CDS
