# Supplementary figures and images for: Comparative Genome Analysis of 33 Chlamydia Strains Reveals Characteristic Features of Chlamydia Psittaci and Closely Related Species
Source: Pathogens. 2020 Oct 28;9(11):899. doi: 10.3390/pathogens9110899 (PMC7694038; doi:10.3390/pathogens9110899)

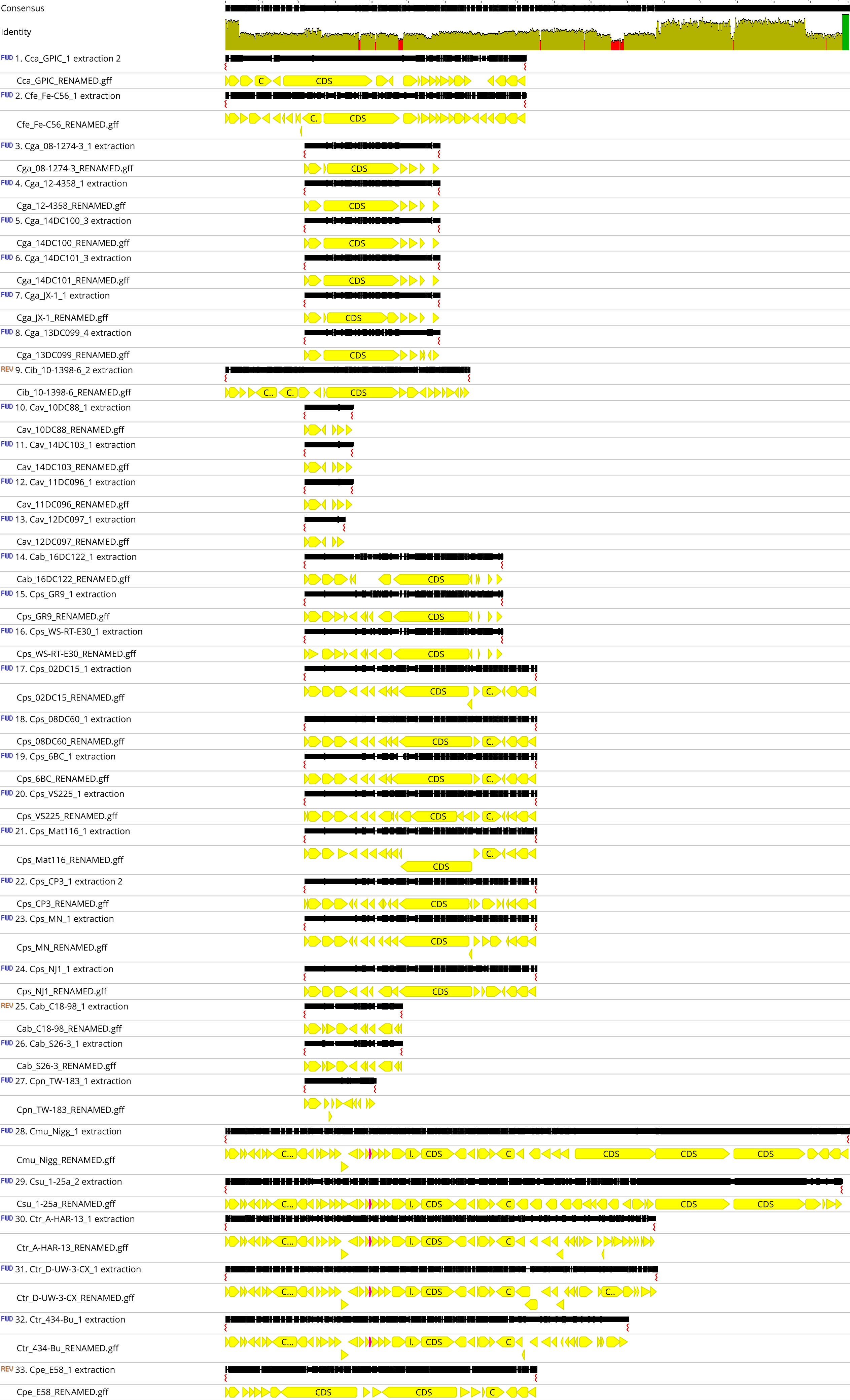

Supplement: Supplementary file 1 [file pathogens-09-00899-s001.zip › Hölzer et al_FigureS2_PZ_alignment_ClustalW.pdf]
